# Structural and Antimicrobial Characterization of Porcine and Fish Gelatin Hydrogels Photochemically Crosslinked with Menadione Sodium Bisulfite

**DOI:** 10.3390/gels12070629

**Published:** 2026-07-15

**Authors:** Vladislav Abramov, Yuriy F. Zuev, Mariya A. Klimovitskaya, Polina V. Skvortsova, Galina J. Yakovleva, William Kurdy, Olga N. Ilinskaya

**Affiliations:** 1 Kazan Institute of Biochemistry and Biophysics, FRC Kazan Scientific Center of RAS, Lobachevsky Str. 2/31, 420111 Kazan, Russia; yufzuev@mail.ru (Y.F.Z.); mklimovitskaya@mail.ru (M.A.K.); skvpolina@gmail.com (P.V.S.); 2 Institute of Fundamental Medicine and Biology, Kazan Federal University, Kremlevskaya St. 18, 420008 Kazan, Russia; yakovleva_galina@mail.ru (G.J.Y.); william.m.kurdy@hotmail.com (W.K.)

**Keywords:** gelatin hydrogel, menadione sodium bisulfite, photochemical crosslinking, antimicrobial activity, ATR-FTIR spectroscopy, NMR spectroscopy, wound dressing

## Abstract

Gelatin-based hydrogels are promising matrices for wound management, but their direct application is constrained by insufficient structural stability and lack of intrinsic antimicrobial activity. Porcine and fish gelatin hydrogels were photochemically crosslinked with menadione sodium bisulfite (MSB), a water-soluble hemostatic derivative of vitamin K3, and characterized by ATR-FTIR and ^1^H NMR spectroscopy, together with antimicrobial testing against *Staphylococcus aureus*, *Candida albicans*, *Escherichia coli*, and *Salmonella enterica*. FTIR analysis showed that MSB crosslinking retards the thermal disruption of collagen-like triple helices in both gelatins, with the effect being more pronounced in porcine gelatin owing to its higher imino acid content and more developed collagen-like network. NMR measurements confirmed that crosslinking increases the bound-water fraction approximately threefold in porcine and twofold in fish gelatin, while the bulk water mobility stays unchanged. MSB crosslinking enhanced antimicrobial activity against *S. aureus* and *C. albicans* by up to 5.6-fold relative to non-crosslinked controls and additionally conferred activity against *E. coli*, while *S. enterica* remained resistant in all variants. MSB, thus, simultaneously serves as a structural crosslinker and imparts intrinsic antimicrobial activity to the resulting hydrogels, making them a promising basis for multifunctional wound-healing materials.

## 1. Introduction

Chronic wounds remain one of the major challenges of modern healthcare, with wound infections and the formation of bacterial biofilms significantly slowing the healing process and increasing the risk of complications [1,2,3]. In the context of growing microbial resistance to antibiotics, there is an increasing demand for wound dressings that provide not only barrier protection and maintenance of a moist wound environment, but also intrinsic antimicrobial activity [4,5]. Recent work on hydrogel wound dressings increasingly combines several functions in one material rather than relying on a single effect. For instance, a bilayer hydrogel has been designed to quickly seal a bleeding wound while its outer layer serves as a physical barrier against external damage, demonstrating that hemostatic action and mechanical protection can coexist in the same dressing [6]. Self-healing hydrogel networks follow a similar logic. These materials restore their own structure after mechanical damage and simultaneously carry antimicrobial, antioxidant, and pro-angiogenic functions that target the multiple problems present in a chronic wound [7]. Together these examples point to a shared expectation for modern wound dressings, namely that such a material should offer both structural resilience and intrinsic biological activity.

Beyond these individual examples, recent reviews have converged on a shared set of performance criteria that define what a modern wound-healing hydrogel should deliver [8,9,10]. An advanced dressing is now expected to combine several complementary functions within a single material rather than to act as a passive barrier, so that it can suppress microbial colonization through intrinsic or loaded antimicrobial activity, sustain a moist yet well-managed wound interface, permit gaseous exchange, and remain mechanically resilient and adherent under the movement of the healing site [8,9]. In parallel, growing emphasis is placed on antioxidant and anti-inflammatory action, hemostatic capacity, and dependable biocompatibility and biodegradability, together with responsive behavior that lets the material adapt to the changing wound microenvironment [9,10]. Framed in this way, multifunctionality is no longer an added benefit but a baseline expectation, and the value of a new dressing rests on how many of these requirements it can satisfy at the same time without compromising safety.

These requirements are increasingly being met with protein-based biopolymer networks, which offer intrinsic biological activity alongside tunable structural performance and have therefore become a favored basis for functional wound dressings [11,12]. Among the biopolymers considered as a basis for such materials, gelatin occupies a special place owing to its biocompatibility, biodegradability, hemostatic action, and structural similarity to extracellular matrix components [13,14,15,16]. Gelatin, as a product of partial denaturation of collagen, retains the arginine-glycine-aspartic acid sequences that mediate fibroblast adhesion and proliferation through interaction with cell-surface integrins [17,18,19]. The hemostatic properties of gelatin have been employed in clinical practice for over seven decades in the form of Gelfoam (Pfizer, New York, NY, USA) and Surgifoam (Ethicon, Soborg, Denmark) sponges, as well as the injectable preparations Surgiflo (Ethicon, Soborg, Denmark) and Floseal (Baxter Healthcare Corporation, Hayward, CA, USA) [20].

Porcine and bovine skin and bones remain the conventional sources of medical-grade gelatin. Fish gelatin, recovered as a by-product of fish processing, has meanwhile attracted growing attention because it satisfies religious and dietary restrictions and allows fishery waste to be valorized [21,22,23]. However, gelatin from cold-water fish differs from mammalian gelatin in several physicochemical characteristics. The limited content of proline and hydroxyproline in cold-water fish gelatin leads to a lower melting point and gelation temperature, as well as weaker gel strength. Consequently, its application at physiological temperatures is restricted [24,25,26,27].

To improve the stability and thermal resistance of gelatin matrices, the physical, chemical and enzymatic approaches are employed, including blending with polysaccharides, covalent crosslinking with low-molecular-weight agents and enzymatic modification with transglutaminase [28,29,30,31,32]. Among classical chemical crosslinkers, glutaraldehyde, genipin, and carbodiimides are widely used. Their application is, however, limited by the cytotoxicity of residual glutaraldehyde molecules, the high cost and intense tissue staining by genipin, the effect of carbodiimides on cellular recognition of the matrix and the rate of material degradation [33,34,35,36]. Photochemical strategies have gained particular attention in recent years, ranging from UV or visible-light crosslinking of methacrylated gelatin to riboflavin-mediated photoinitiation [37,38,39]. Residual methacrylate monomer and the shallow tissue penetration of visible light, however, continue to drive the search for alternative photoinitiating systems [40]. One such alternative is menadione sodium bisulfite (MSB), a clinically used hemostatic agent that represents a water-soluble form of vitamin K3 [41,42]. It has recently been shown that MSB can initiate radical crosslinking of protein chains under UV irradiation through a type I photoreaction. Upon UV excitation, MSB is promoted to a reactive triplet state, in which the carbonyl oxygen behaves as a hydrogen-abstracting radical acceptor, analogous to other quinone and ketone-based photoinitiators. This excited MSB preferentially abstracts hydrogen atoms from the α-carbon positions of the gelatin backbone and side chains, generating carbon-centered protein radicals. Recombination of these radicals across different peptide chains forms new covalent C–C bonds, which constitute the crosslinks [43,44,45]. In this process MSB acts as a photoinitiator and is not incorporated into the gelatin backbone to any significant extent, remaining in the hydrogel matrix in a free, physically entrapped form [45].

In addition to structural changes induced by crosslinking, the incorporation of MSB into the gelatin matrix is expected to impart the antimicrobial activity to resulting hydrogels. Since gelatin arises from partial hydrolysis of collagen, it may inherit the internal antimicrobial peptide segments already described within several collagen types, active against both Gram-positive and Gram-negative bacteria [46,47]. Consequently, the concentrated gelatin matrices may retain these bioactive fragments.

Menadione and its derivatives exhibit antimicrobial activity mediated by two fundamentally different pathways. Under UV or blue light, MSB behaves as a photosensitizer, producing reactive oxygen species capable of inactivating both bacterial groups [45,48]. Conversely, the antimicrobial activity of menadione also persists in the dark. As a redox-active naphthoquinone, menadione accepts electrons from oxidoreductases of the bacterial respiratory chain and transfers them to molecular oxygen, generating a superoxide anion directly in cytoplasm [49,50]. In addition, menadione and other quinones can bind to thiol groups in bacterial proteins and low-molecular-weight cellular antioxidants. Such binding disrupts enzymatic function and depletes the antioxidant defense of bacteria, reducing their viability [51,52]. Furthermore, under dark conditions, pure menadione induces bacterial membrane permeabilization via a non-oxidative mechanism, leading to damage of the membrane barrier function and increased susceptibility of both Gram-positive and Gram-negative cells to antibiotics [53]. In line with the latter observation, recent reports have demonstrated synergy between menadione and several antibiotic classes against drug-resistant Gram-negative pathogens. Pairing menadione with colistin has been shown to compromise the outer membrane, reduce the proton-motive force and intracellular ATP levels, and stimulate reactive oxygen species production, ultimately leading to extensive bacterial killing in both colistin-susceptible and colistin-resistant strains [54]. MSB combines the ability to initiate covalent crosslinking in proteins with pronounced intrinsic antimicrobial activity, making it a promising basis for the design of multifunctional biomaterials.

Building on these considerations, the main goal of the present work is to characterize the intrinsic antimicrobial activity that MSB imparts to porcine and fish gelatin hydrogels, since the dual role of MSB as both a photocrosslinker and an antimicrobial agent has not yet been examined for these systems. Alongside this antimicrobial characterization, we carried out new structural measurements by ^1^H NMR spectroscopy and ATR-FTIR spectroscopy that probe the state of confined water and the thermal behavior of the collagen-like helices. These measurements complement the structural characterization of the identical MSB-crosslinked porcine and fish gelatin hydrogels that we reported earlier by rheology, scanning electron microscopy and dielectric spectroscopy [55]. The present study is therefore limited to the antimicrobial and structural characterization of these hydrogels, whereas their detailed biological and mechanical evaluation under wound-mimicking conditions remains a separate, ongoing line of investigation.

## 2. Results and Discussion

### 2.1. FTIR Study of Protein and Water Molecular Properties in Hydrogels

**Figure 1:**
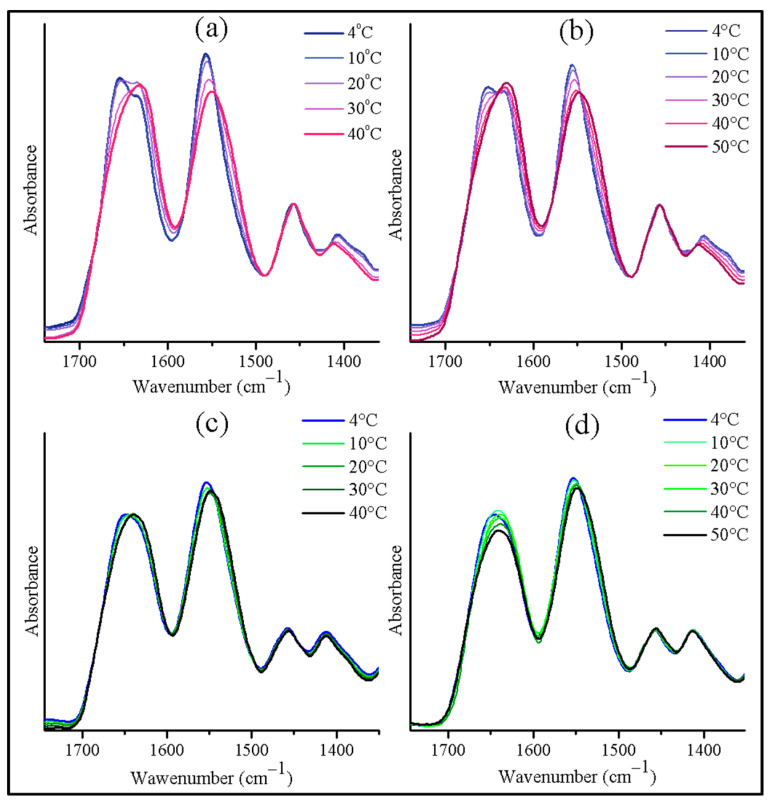
Amide-I (C=O) and Amide-II (N-H) absorption regions of porcine (**a**,**b**) and fish (**c**,**d**) gelatin hydrogels before (**a**,**c**) and after (**b**,**d**) crosslinking.

FTIR spectra were obtained for non-crosslinked and crosslinked porcine (PG) and fish (FG) gelatin hydrogels upon stepwise heating from 4 to 50 °C. All spectra were normalized to intensity of the CH_2_ bending band at 1450 cm^−1^ (Figure 1). The complete spectra covering the entire measured range are provided in the Supplementary Materials (Figure S1). Upon heating, the intensity of the 1660 cm^−1^ component in the Amide-I region, assigned to C=O vibrations of the triple helix [56,57], progressively decreases in all four systems, with the change being substantially more pronounced in porcine than in fish gelatin. In porcine gelatin, this decrease is accompanied by a monotonic increase in intensity near 1622 cm^−1^, reflecting the increasing contribution of carbonyl groups in disordered chains. In fish gelatin, the corresponding change near 1622 cm^−1^ is weaker and non-monotonic, reaching a maximum at about 20 °C and decreasing upon further heating. This behavior reflects the lower melting temperature of the triple-helix structure in fish gelatin, whose helix-to-coil transition is already nearly complete at moderate temperatures. The maximum of the Amide-II band shifts to longer wavelengths in all four samples, which is associated with the rupture of N–H(Gly)···O=C(Pro) hydrogen bonds of the triple helix, with the shift being larger in porcine gelatin in agreement with its higher initial triple-helix content [58].

**Figure 2:**
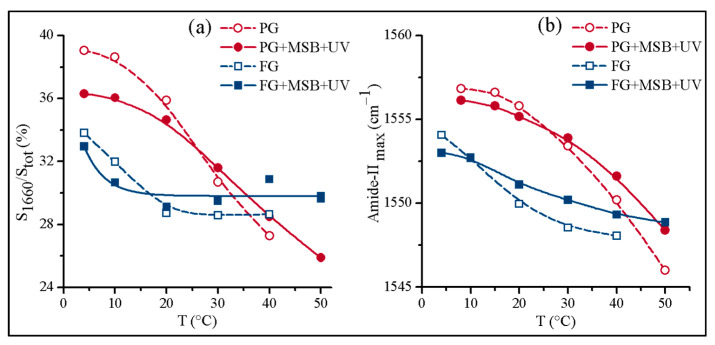
Temperature dependence of triple-helix fraction S_1660_/S_tot_ (**a**) and the position of Amide-II band maximum (**b**) in hydrogels.

The triple-helix content in the protein matrix was estimated from deconvolution of the Amide-I band into six Gaussian components centered at 1618, 1630, 1642, 1660, 1680 and 1692 cm^−1^, each corresponding to a distinct C=O group within the Gly-Pro-Hyp repeat of the gelatin chain [27,59,60]. A representative example of this deconvolution is shown in the Supplementary Materials (Figure S2). The relative contribution of the 1660 cm^−1^ component to the total band intensity, S_1660_/S_tot_, was used as a measure of helical content. The temperature dependences of S_1660_/S_tot_ and of the Amide-II band maximum position for all four systems are shown in Figure 2.

Porcine gelatin displays a markedly higher helical content than fish gelatin at 4 °C, consistent with its greater proportion of imino acid residues, which stabilize the collagen-like arrangement [61]. In both gelatins, the helical content of crosslinked sample at 4 °C is somewhat lower than that of the non-crosslinked one, reflecting the local distortion of helix packing that accompanies radical crosslinking under UV irradiation. With increasing temperature, S_1660_/S_tot_ decreases monotonically in all four systems, and the decrease is less pronounced in the crosslinked samples than in the corresponding non-crosslinked ones. The stabilizing effect of crosslinking is more evident in porcine gelatin, which initially has a more developed collagen-like structure. This agrees with the rheology of the identical systems [55], where MSB crosslinking produced a pronounced upward shift in the gel-sol transition temperature for porcine gelatin while the effect for fish gelatin was markedly weaker. The storage modulus G′ of porcine gelatin at 4 °C increased approximately 2.5-fold upon crosslinking, and the elastic gel state was retained at elevated temperature [55].

Notably, the 1660 cm^−1^ component does not vanish completely even once the protein is fully denatured [60], because carbonyl groups belonging to non-imino residues at the X and Y positions of the collagen sequence contribute to this region irrespective of helix conformation. Accordingly, the absolute S_1660_/S_tot_ values overestimate the true triple-helix content, particularly at elevated temperatures, but can be used for comparative analysis of helical content and crosslinking effects.

The temperature dependence of the Amide-II band maximum position (Figure 2b) supports the conclusions drawn from the S_1660_/S_tot_ data. The temperature-induced band shift in the crosslinked samples is about 1.5 times smaller than in the non-crosslinked ones, indicating stabilization of helical elements by a covalent network. A noticeable shift in the Amide-II band maximum in porcine gelatin begins above 20 °C, while in fish gelatin it already appears above 10 °C, which reflects the lower thermal stability of fish hydrogel. As previously demonstrated for crosslinked porcine gelatin and native rat-tail collagen [58], the position of the Amide-II maximum after reaching the plateau of full denaturation is about 1541 cm^−1^. The Amide-II band position in the studied gelatins at 50 °C does not reach this value, indicating that denaturation at this temperature remains incomplete.

**Figure 3:**
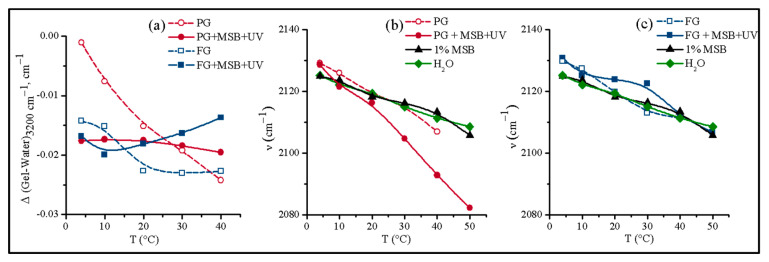
Temperature dependence of difference signal at 3200 cm^−1^ (**a**), maximum position of water band 2130 cm^−1^ for pure water, 1% MSB solution and non-crosslinked and crosslinked hydrogels of porcine (**b**) and fish (**c**) systems.

To assess the state of the gelatin hydration shell, the OH stretching difference spectra were used, specifically the value of difference signal at 3200 cm^−1^ normalized to the intensity of the CH_2_ band. This region contains OH vibrations of water molecules involved in three or four hydrogen bonds, a signature of unperturbed bulk liquid water, so a negative difference reflects a decrease in their fraction in the presence of the protein matrix. Temperature dependences of this quantity for both gelatins are shown in Figure 3a.

The non-crosslinked porcine gelatin hydrogel shows a monotonic increase in the absolute value of negative difference signal with temperature, whereas in the crosslinked hydrogel the same dependence is much less pronounced. The difference is associated with restriction of peptide chain mobility by a covalent network. Heating of the non-crosslinked porcine hydrogel is accompanied by unfolding of helical fragments and dispersion of individual chains, which maximizes the accessibility of protein polar groups to water. In the crosslinked hydrogel, the gelatin chain swelling is hampered, and the accessibility of polar sites only changes weakly upon heating. In the fish gelatin hydrogels, both systems show weakly varying signals, reflecting the initially less dense organization of the polymer network.

The additional information about the state of water is provided by position of the combination band near 2130 cm^−1^, arising from the sum of bending and librational OH motions [58]. Unlike stretching and bending vibrations, the librational motions are sensitive to the energy exchange between neighboring water molecules and therefore characterize the collective dynamics of water molecules within the hydrogen-bond network [62]. A red shift in the band maximum upon heating reflects the weakening of the network and its thermal expansion. The corresponding dependences for pure water, a 1% MSB solution, and the non-crosslinked and crosslinked hydrogels of both gelatins are shown in Figure 3b,c.

In the crosslinked porcine gelatin hydrogel, the temperature-induced shift in combination band maximum substantially exceeds that in pure water, while the non-crosslinked hydrogel occupies an intermediate position. By restricting the mobility of peptide fragments, the covalent crosslinking in porcine gelatin additionally perturbs the collective dynamics of water, and this perturbation grows with heating. In fish gelatin, the shift in maximum in crosslinked hydrogel is close to that in pure water, and at some temperatures even lower, indicating that covalent crosslinking in fish gelatin practically does not perturb collective dynamics of surrounding water. The difference between the two gelatins reflects different structural organizations of the networks formed upon crosslinking. In porcine gelatin, with its high content of imino acid residues, a more developed and rigid framework is formed, which impedes collective redistribution of water hydrogen bonds, whereas in the fish gelatin the network remains comparatively loose and does not restrict librational motions of water molecules. This structural difference is consistent with the earlier characterization of the identical MSB-crosslinked systems [55], where rheology, scanning electron microscopy and dielectric spectroscopy independently revealed a stronger and more ordered porcine network together with a more mobile, weakly coupled bound-water population in fish gelatin. The morphology of crosslinked porcine hydrogel showed a denser network built of numerous small pores separated by thin partitions, while the crosslinked fish hydrogel formed larger, more regular oval pores with thicker partitions, indicating a more ordered but mechanically weaker organization. The morphological contrast matches the difference in the properties of confined water inferred here from the FTIR data.

In summary, the MSB crosslinking slows the thermal disruption of collagen-like helices in both porcine and fish gelatin hydrogels and at the same time affects the state of water differently depending on the protein origin. In porcine gelatin, which initially has a more developed collagen-like structure, the covalent network stabilizes the hydration shell and simultaneously enhances perturbation of the collective dynamics of water upon heating, reflecting formation of the dense, rigid framework. In fish gelatin, in which the initial content of ordered helical fragments is lower, the network formed upon crosslinking remains loose, and its influence on both the hydration shell and the collective dynamics of water is significantly weaker. Overall, the MSB acts as a versatile crosslinking agent for gelatins of different origins, but structural characteristics of the resulting network and its interaction with the solvent depend substantially on the protein amino acid composition.

### 2.2. Dynamic State of Water in Hydrogels via NMR Experiments

**Figure 4:**
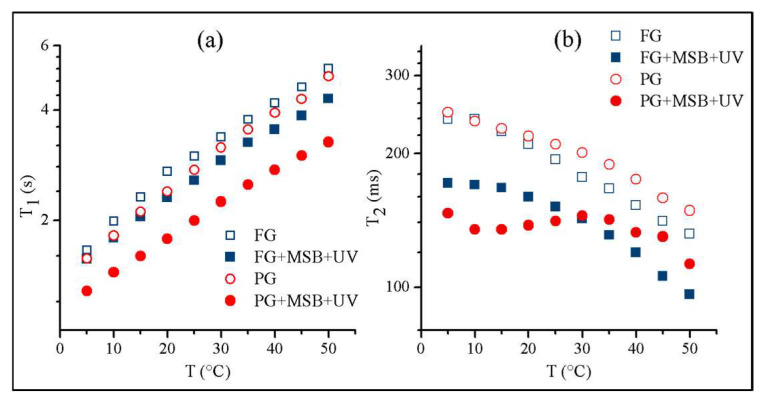
Temperature dependences of *T*_1_ (**a**) and *T*_2_ (**b**) of water in fish and porcine gelatin hydrogels in non-crosslinked and crosslinked states.

Another perspective on the hydrogels’ properties was obtained in the NMR experiments, in which the spin-lattice (*T*_1_) and spin-spin (*T*_2_) relaxation times together with the self-diffusion coefficient (*D*) of water were measured, since the dynamics of water serves as a structural marker of the polymer network and of the nature of its interaction with the solvent [63]. Water in hydrogels exists in two dynamically distinct states [58]. One part of its molecules behaves as free water, while the other is bound to the protein matrix and has restricted mobility. Since the exchange between these fractions is sufficiently fast on the time-scale of NMR experiments, the experimentally measured values of *T*_1_, *T*_2_, and *D* are averaged and reflect the weighted contribution of both fractions [64,65]. The corresponding temperature dependences of *T*_1_ and *T*_2_ are shown in Figure 4.

Under fast exchange conditions, the spin-lattice relaxation of water is described by the two-fraction model [66]:

(1) $$\frac{1}{T_{1}}=\frac{p}{T_{1bound}}+\;\frac{1-p}{T_{1free}},$$

with *p* denoting the fraction of bound water, while *T*_1bound_ and *T*_1free_ stand for the spin-lattice relaxation times of the bound and free fractions, respectively. In both gelatins, the crosslinking by MSB decreases *T*_1_ over the entire temperature range, which reflects an increased fraction of bound water and is in line with the FTIR observation of enhanced perturbation of collective water dynamics in the crosslinked porcine matrix. To estimate the bound-water fraction, the data were processed using Equation (1) with the first-approximation assumption $T_{1bound}=0.1\cdot T_{1free}$ [67], with the $T_{1free}$ values for pure water at the corresponding temperatures interpolated from the tabulated data of [68]. Although the absolute values of these fractions depend on the chosen approximation for $T_{1bound}$, the estimates are reliable for comparative analysis, as the same assumption was applied uniformly to all samples. The complete set of $T_{1free}$ values, measured *T*_1_, and resulting *p* used to obtain these estimates is provided in the Supplementary Materials (Table S1). The resulting estimates show that MSB crosslinking increases the bound-water fraction in fish gelatin approximately twofold, from 2% to 4% at 25 °C, and in porcine gelatin approximately threefold, from 3% to 9% at the same temperature. The more pronounced effect in porcine gelatin is consistent with its higher imino acid content and the formation of a more developed network with a larger number of collagen-like triple helices.

The spin-spin relaxation is sensitive not only to molecular mobility of water but also to chemical proton exchange between water and protein [69]. Since this exchange accelerates with increasing temperature, *T*_2_ naturally decreases upon heating for all studied samples. The MSB crosslinking additionally reduces *T*_2_ in both gelatins, and this effect is stronger in porcine gelatin, being in good agreement with the *T*_1_ data and confirming the enhanced interaction of water with the protein matrix upon formation of a covalent network.

**Figure 5:**
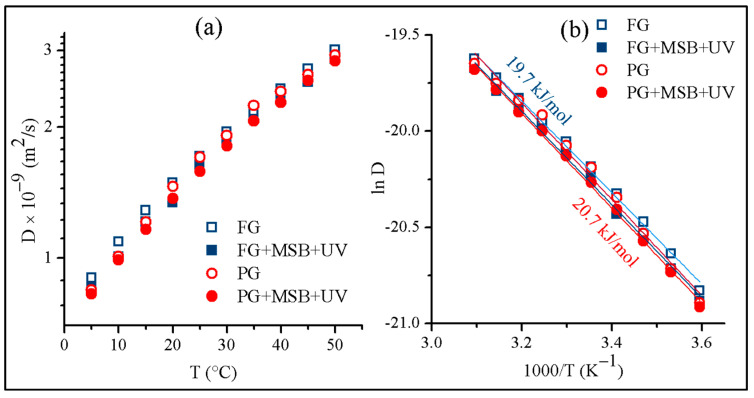
Temperature dependence (**a**) and Arrhenius plot (**b**) of water self-diffusion coefficient *D* in fish and porcine gelatin hydrogels in non-crosslinked and crosslinked states.

Figure 5 shows the temperature dependence of the self-diffusion coefficient *D*, together with its representation in Arrhenius coordinates used to determine the activation energy of water diffusion.

The self-diffusion coefficient of water directly characterizes its diffusional mobility. The MSB crosslinking slightly decreases *D* in both gelatins, with the effect being somewhat stronger in porcine than in fish gelatin. The fact that the decrease in *D* remains modest indicates that the bulk water in hydrogel preserves its properties close to those of pure water, while the restriction of mobility is localized to a small bound-water fraction that experiences spatial hindrance within the dense crosslinked network. The activation energy of water diffusion, determined from the temperature dependence of *D* in Arrhenius coordinates, was found to be similar for all four systems, amounting to about 20 kJ/mol. This value slightly exceeds the activation energy of pure water (17.8 kJ/mol [70]), reflecting additional spatial obstacles to diffusion created by the polymer network of hydrogels.

Comparison between the two gelatins reveals a coherent picture across all three parameters. In the crosslinked hydrogels, the values of *T*_1_, *T*_2_ and *D* in porcine gelatin are systematically lower than in the fish gelatin over the entire studied temperature range, indicating a larger bound-water fraction and a more pronounced restriction of its mobility in the porcine system. In the non-crosslinked gels, the difference between the two gelatins is small, reflecting a similar state of water in the physical networks of both gelatins before the introduction of covalent crosslinks.

### 2.3. Antimicrobial Activity

In addition to the structural characterization, the antimicrobial activity was evaluated for both initial gelatin solutions used for hydrogel formation and the resulting gel films against *Salmonella enterica*, *Staphylococcus aureus*, *Escherichia coli* and *Candida albicans*.

**Figure 6:**
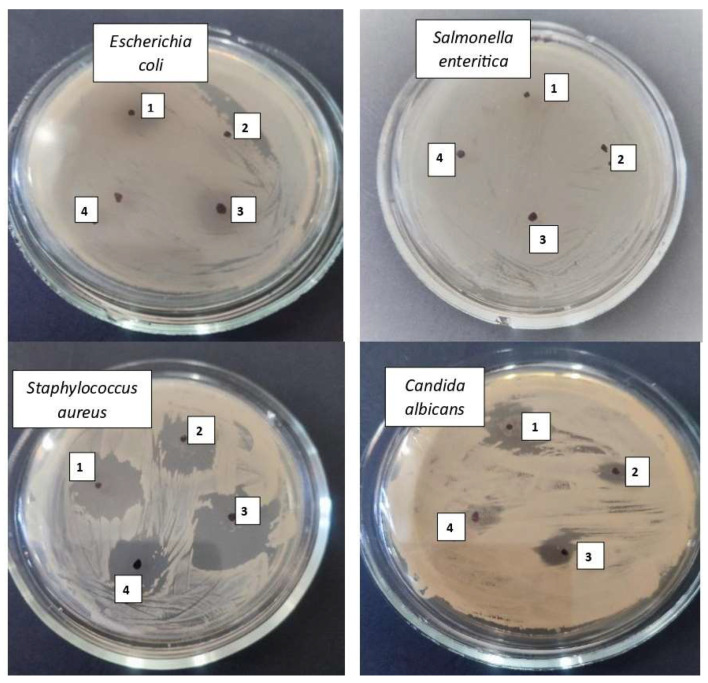
Inhibition zones induced by gelatin hydrogel samples: 1—crosslinked PG; 2—non-crosslinked PG; 3—crosslinked FG; 4—non-crosslinked FG.

It was found that the non-crosslinked gelatin samples showed no activity against Gram-negative bacteria (Figure 6). The crosslinking of porcine and fish gelatins with MSB results in the appearance of minor zones of inhibition in *E. coli*, while *S. enterica* remained unaffected in all variants. The most sensitive to the antimicrobial action of hydrogels were Gram-positive bacteria and yeast, namely microorganisms that have polysaccharides on their surface and lack an outer lipid membrane.

**Figure 7:**
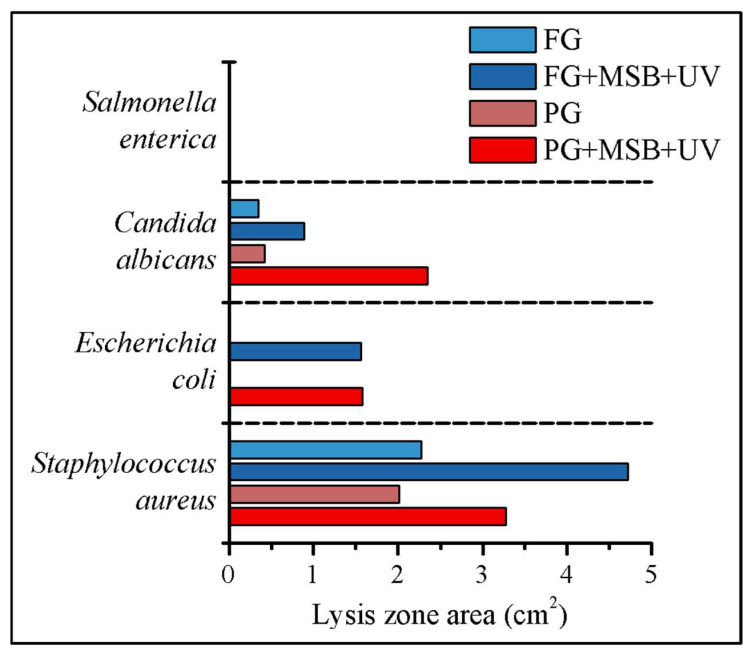
Inhibition zone areas of microbial cultures for non-crosslinked and MSB-crosslinked hydrogels based on PG and FG.

The growth of Gram-positive *S. aureus* was significantly inhibited by hydrogels containing MSB, and even the non-crosslinked hydrogels showed this effect, although it was reduced by factors of 1.6 and 2.0 for porcine and fish gelatin, respectively. MSB-crosslinked hydrogels also had the most effective antimicrobial action against yeast, while the activity of the crosslinked porcine hydrogel was 5.6-fold higher than that of the non-crosslinked porcine sample and 2.5-fold higher for the fish gelatin sample (Figure 7).

The similar levels of activity of non-crosslinked gelatin samples of different origin indicate that the observed effect is determined by general properties of concentrated protein matrix rather than by the gelatin source. This is likely associated with the presence of short peptide fragments derived from the antimicrobial regions of collagen [46,47], as well as with the reduced water activity and the unfavorable environment for bacterial growth that arises in the contact zone of highly concentrated gelatin matrix [71]. The absence of activity of non-crosslinked samples against *E. coli* is most probably due to the outer membrane with its dense lipopolysaccharide layer, being a characteristic of Gram-negative bacteria and serving as a barrier to most external stresses [15,72].

The enhancement of activity following the photoinduced crosslinking, and the appearance of growth inhibition for *E. coli*, indicate that MSB itself contributes to the antimicrobial action of crosslinked hydrogels, as it can perturb bacterial homeostasis through several mechanisms described in the literature [49,50,51,52,53]. A similar antimicrobial effect has been previously reported for other MSB-based materials, including photoactive water-soluble derivatives of vitamin K3 and gelatin coatings [43,45].

The differences between the MSB-crosslinked hydrogels based on porcine and fish gelatins are small and depend on the type of microorganism. The fish hydrogel is somewhat more active against *S. aureus* and the porcine hydrogel against *C. albicans*, while the activities of both samples against *E. coli* are nearly identical. These differences are likely related to the different structural organizations of the polymer networks, consistent with the results of FTIR and NMR experiments. The looser network of fish hydrogel may favor faster diffusion of MSB toward the contact surface, which particularly affects the action against Gram-positive *S. aureus*. The denser framework of porcine hydrogel, in turn, presumably retains the active component within the matrix for longer times, which may play a role in the action on *C. albicans* cells with their thicker, chitin-rich cell wall. Overall, the antimicrobial response of both gelatin types after MSB crosslinking is comparable, indicating the universality of the proposed approach for obtaining functional hydrogels.

The analysis of the microbial growth inhibition by gelatin-based films revealed a distinct trend. In general, all non-crosslinked films were ineffective (Table 1). The irradiation of these films for 20 min with UV light and their immediate application to the surface of cultures in Petri dishes did not induce antimicrobial activity, except for the fish gelatin variant, which caused a minor growth inhibition of the most sensitive strain *S. aureus*. This effect may tentatively be attributed to UV-induced partial cleavage of the fish gelatin polypeptide chain, which could expose cryptic antimicrobial peptide sequences as reported for fish collagen-derived fragments [73,74]. However, weak activity against *E. coli* was detected for the fish film containing MSB, but not for analogous porcine film.

The UV irradiation did not enhance antimicrobial activity of films containing MSB. Storing the films for 24 h after UV irradiation did not change their antimicrobial activity. As in the previous experiments with liquid gelatin samples, *E. coli* was the most resistant to their antimicrobial action. MSB itself inhibited the growth of studied microorganisms, indicating that its inclusion in gelatin films contributed to antimicrobial effect.

**Figure 8:**
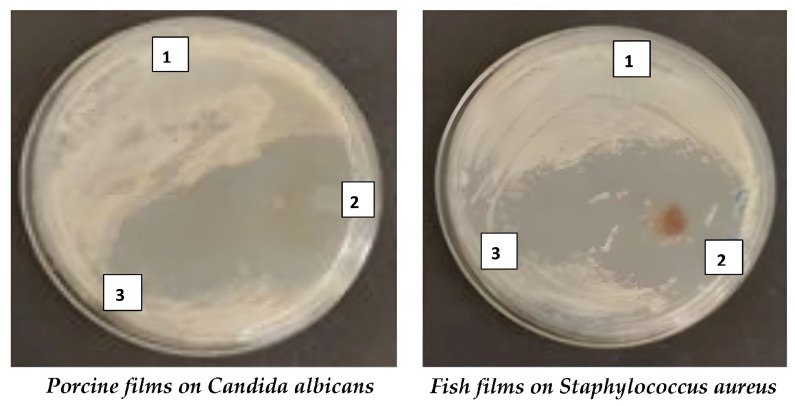
Antimicrobial activity of gelatin films against *C. albicans* and *S. aureus*: 1—films without MSB; 2—MSB-containing films after UV irradiation; 3—MSB-containing films without UV irradiation.

To illustrate the antimicrobial action of MSB-containing gelatin films, Figure 8 presents examples of growth inhibition of *Staphylococcus* and yeast cultures. Non-crosslinked films do not exhibit an antimicrobial effect, whereas the incorporation of MSB in their composition causes the cultures’ growth inhibition regardless of UV irradiation. No fundamental difference was observed between porcine and fish gelatin films. However, immediately after irradiation, the porcine gelatin film containing MSB exhibited no antimicrobial activity against *E. coli*, whereas the same film containing fish gelatin exhibited an antimicrobial effect. This difference is likely due to the properties of gelatins of different origins.

## 3. Conclusions

The present work has examined how photoinduced crosslinking with MSB modifies the structural organization of porcine and fish gelatin hydrogels and whether these structural differences are reflected in their antimicrobial activity. The FTIR analysis of the Amide-I and Amide-II frequency bands has shown that the MSB crosslinking slows thermal collapse of collagen-like triple helices in both gelatin hydrogels, with the stabilizing effect being more pronounced in porcine gelatin, which is initially richer in imino acid residues. The water-related vibrational signals at 3200 cm^−1^ and around 2130 cm^−1^ indicate that the MSB-crosslinked porcine hydrogel forms a denser polymer framework that perturbs the hydration shell and collective dynamics of water more strongly than its fish-gelatin counterpart, whose network remains comparatively loose.

The NMR relaxometry and self-diffusion measurements yield a consistent picture. The MSB crosslinking reduces *T*_1_ and *T*_2_ of water and increases the bound-water fraction in both gelatins, an effect roughly two-to-three-fold stronger for porcine gelatin. Meanwhile, the self-diffusion coefficient of water decreases only moderately and the activation energy of diffusion remains close to that of pure water, indicating that the polymer network does not impede the mobility of bulk water within the hydrogel and acts selectively on the bound-water fraction in immediate contact with the gelatin chains. The preservation of bulk water mobility within the crosslinked network, combined with enhanced binding of water to protein chains, is consistent with the properties required for wound-contact hydrogel materials.

The antimicrobial activity of obtained materials, evaluated against *Staphylococcus aureus*, *Candida albicans*, *Escherichia coli* and *Salmonella enterica*, follows a coherent pattern. Non-crosslinked hydrogels show moderate inhibition of *S. aureus* and *C. albicans* only, while photoinduced crosslinking with MSB substantially enhances the antimicrobial response and confers activity against *E. coli*. The differences between MSB-crosslinked hydrogels of porcine and fish origin are small and microorganism-specific, with the looser fish-gelatin network facilitating more rapid MSB release at the contact surface and showing slightly higher activity against *S. aureus*, while the denser porcine framework retains MSB longer and is more effective against *C. albicans*. *S. enterica* remains resistant in all variants. The same general trends were observed for thin gelatin films, where the presence of MSB was the determining factor for antimicrobial activity.

Taken together, these results establish MSB as a versatile photocrosslinking agent for gelatins of different origins that simultaneously imparts intrinsic antimicrobial activity to the resulting hydrogels and films, with demonstrated efficacy against *S. aureus*, *C. albicans* and *E. coli*. The combination of structural stabilization with antimicrobial activity, together with the hemostatic properties of MSB itself, makes the developed materials a promising basis for the further development of multifunctional wound-dressing materials. Their translation toward practical use will require a dedicated biological and functional evaluation extending beyond the present structural study, including the cytocompatibility of the matrices toward skin cells, their mechanical performance and fluid-handling behavior under physiological conditions, the release kinetics and degradation profile, and a quantitative assessment of antimicrobial efficacy against both planktonic and biofilm-associated pathogens benchmarked against established dressings.

## 4. Materials and Methods

### 4.1. Materials

Two gelatin types served as protein matrices in this work. Porcine gelatin (Type A, 300 Bloom, food grade, Sigma-Aldrich, product G2500, St. Louis, MO, USA) represented the mammalian source, whereas fish gelatin was prepared in-house through acid extraction from the skin of Atlantic cod [75]. Photocrosslinking of both gelatins was induced using MSB (Sigma-Aldrich, product M5750, St. Louis, MO, USA) as the crosslinking agent. Ultrapure water was produced with a HyperPureX EUS 13 system (Hyperpurex Instrument Technology, Shanghai, China).

### 4.2. Preparation of Hydrogels

Hydrogels crosslinked with MSB were obtained according to a protocol established in our earlier work [58], with minor adaptation. A weighed quantity of gelatin was allowed to swell overnight in ultrapure water at room temperature, then dissolved by stirring on a thermal shaker (Joan Lab, Huzhou, China) for 1 h at 50 °C and 1000 rpm, giving a 12.5% (*w*/*v*) gelatin stock solution. Separately, a 5% (*w*/*v*) MSB stock solution was prepared in ultrapure water and combined with the warm gelatin solution to reach final concentrations of 10% gelatin and 1% MSB, after which the mixture was stirred for 1 h at 40 °C.

The gelatin-MSB mixture was cast into a 90 mm diameter mold to obtain a 1 mm thick film. Gelation was allowed to proceed overnight at 4 °C, after which the samples were frozen for 18 h at −40 °C and thawed for 6 h at 4 °C. Photocrosslinking was carried out by exposing the mold to UV light for 20 min in a UVA irradiation chamber (350 nm, 4 W) held at 4 °C. Non-crosslinked control gels (10% gelatin) were obtained by an identical procedure, with the MSB addition and UV exposure steps omitted.

### 4.3. FTIR Spectroscopy

ATR-FTIR spectroscopy was applied to probe the secondary structure of gelatin and the hydration state of the hydrogels. Measurements were carried out on an Invenio S FTIR spectrometer (Bruker Optik GmbH, Ettlingen, Germany) fitted with a triple-reflection ZnSe ATR accessory. For each spectrum, 128 scans were co-added at a resolution of 4 cm^−1^ across the range from 4000 to 800 cm^−1^. Gel samples kept at 4 °C were transferred onto the ATR crystal, itself pre-cooled to 4 °C, and spectra were acquired stepwise over a temperature interval from 4 to 50 °C. Contributions from bulk water and atmospheric vapor were subtracted from the raw spectra, and all processing was carried out in OPUS software (version 7.2, Bruker Optik GmbH, Ettlingen, Germany).

### 4.4. NMR Relaxation and Self-Diffusion Measurements

Proton NMR relaxation and self-diffusion of water were measured on a 600 MHz Bruker AVANCE III spectrometer (Bruker BioSpin GmbH, Rheinstetten, Germany) fitted with a triple resonance TBI probe, z-gradients reaching a maximum of 55.7 G cm^−1^, and a BCU05 unit for temperature regulation. Samples were heated stepwise from 5 to 50 °C in increments of 5 °C for all experiments. Self-diffusion coefficients were obtained with a bipolar-gradient stimulated echo sequence (stebpgp1s), sweeping the gradient amplitude from 1 to 48 G/cm at a fixed diffusion time of 50 ms and gradient duration of 1 to 2 ms. Spin-lattice (*T*_1_) and spin-spin (*T*_2_) relaxation times were recorded respectively with the inversion-recovery (t1ir) and Carr–Purcell–Meiboom–Gill (cpmg, *τ* = 1.5 ms) sequences. All resulting data were analyzed in TopSpin (version 3.6, Bruker BioSpin GmbH, Rheinstetten, Germany). Representative examples of the diffusion decay and relaxation fits used to extract *D*, *T*_1_ and *T*_2_ are provided in the Supplementary Materials (Figure S3).

### 4.5. Antimicrobial Activity Assay

The antimicrobial activity was tested against clinical isolates of *Salmonella enterica*, *Escherichia coli*, *Staphylococcus aureus* and *Candida albicans*, provided by the Kazan Research Institute of Epidemiology and Microbiology (Federal Service for Surveillance on Consumer Rights Protection and Human Welfare, Kazan, Russia). These isolates were spread onto Petri dishes containing LB agar for bacteria and Sabouraud agar for yeast. A 10 µL aliquot of each gelatin sample was placed directly on the surface of the resulting microbial lawn, and the plates were incubated for 48 h at 37 °C. Photographs were taken to record inhibition zones around gelatin samples. Inhibition zones areas were calculated using ImageJ software (version 1.48v, National Institutes of Health, Bethesda, MD, USA) [76]. The antimicrobial effect of gelatin films was similarly studied. Since the films varied in area and had uneven edges, the antimicrobial effect could not be expressed in terms of inhibition zone area, and the results were evaluated qualitatively as presence/absence of inhibition zones.

## Figures and Tables

**Table 1:** Antimicrobial activity of gelatin-based films.

Samples	Antimicrobial Activity Against Bacteria and Yeast *			
	*Escherichia* *coli*	*Staphylococcus* *aureus*	*Candida* *albicans*	*Salmonella* *enterica*
PG	−	−	−	−
PG + MSB	+	+	+	−
PG + MSB + UV	+	+	+	−
FG	−	−	−	−
FG + MSB	−	+	+	−
FG + MSB + UV	−	+	+	−
MSB 5%	+	+	+	−
PG immediately after UV	−	−	−	−
PG + MSB immediately after UV	−	+	+	−
FG immediately after UV	−	+	−	−
FG + MSB immediately after UV	+	+	+	−

* (−)—absence of inhibition zone, (+)—presence of inhibition zone.

## Data Availability

All the data used for the analyses in this report are available from the corresponding authors upon reasonable request.

## Supplementary Materials

The following supporting information can be downloaded at https://www.mdpi.com/article/10.3390/gels12070629/s1. Figure S1: Full ATR-FTIR spectra (4000–800 cm^−1^) of non-crosslinked and MSB-crosslinked porcine (PG) and fish (FG) gelatin hydrogels at 20 °C. Spectra are vertically offset for clarity; Figure S2: Deconvolution of the Amide-I band into six Gaussian components at 20 °C for (**a**) PG, (**b**) PG + MSB + UV, (**c**) FG, and (**d**) FG + MSB + UV; Figure S3: Representative fits used to extract water dynamics parameters from NMR data, shown for fish gelatin (FG) at 5 °C: (**a**) diffusion decay, ln(I/I_0_) versus b-factor, fitted with the Stejskal–Tanner equation to obtain the self-diffusion coefficient *D*; (**b**) *T*_1_ relaxation recovery curve, signal intensity versus relaxation delay *τ* (s), fitted with an exponential recovery function; (**c**) *T*_2_ relaxation decay measured by the CPMG pulse sequence, ln(I/I0) versus echo time *τ* (ms), fitted with a mono-exponential decay function; Table S1: Temperature dependence of the free-water spin-lattice relaxation time (*T*1*free*), the measured population-averaged spin-lattice relaxation time (*T*_1_), and the resulting bound-water fraction (*p*) for non-crosslinked and MSB-crosslinked porcine (PG) and fish (FG) gelatin hydrogels.

## Abbreviations

The following abbreviations are used in this manuscript:

PG	Porcine gelatin
FG	Fish gelatin
MSB	Menadione sodium bisulfite
UV	Ultraviolet
