# Supplementary material for: Structural and Antimicrobial Characterization of Porcine and Fish Gelatin Hydrogels Photochemically Crosslinked with Menadione Sodium Bisulfite
Source: Gels. 2026 Jul 15;12(7):629. doi: 10.3390/gels12070629 (PMC13409681; doi:10.3390/gels12070629)
Supplement: Supplementary file 1 [file gels-12-00629-s001.zip › gels-4401793-supplementary.pdf]

gels

Supplementary materials

Structural and Antimicrobial Characterization of Porcine and Fish Gelatin Hydrogels Photochemically Crosslinked with Menadione Sodium Bisulfite

Vladislav Abramov [1,*], Yuriy F. Zuev [1], Mariya A. Klimovitskaya [1], Polina V. Skvortsova [1], Galina J. Yakovleva [2], William Kurdy [2] and Olga N. Ilinskaya [2,*]

[1] Kazan Institute of Biochemistry and Biophysics, FRC Kazan Scientific Center of RAS, Lobachevsky Str. 2/31, 420111 Kazan, Russia
[2] Institute of Fundamental Medicine and Biology, Kazan Federal University, Kremlevskaya St. 18, 420008 Kazan, Russia
* Correspondence: abramovv660@gmail.com (V.A.); ilinskaya_kfu@mail.ru (O.N.I.)

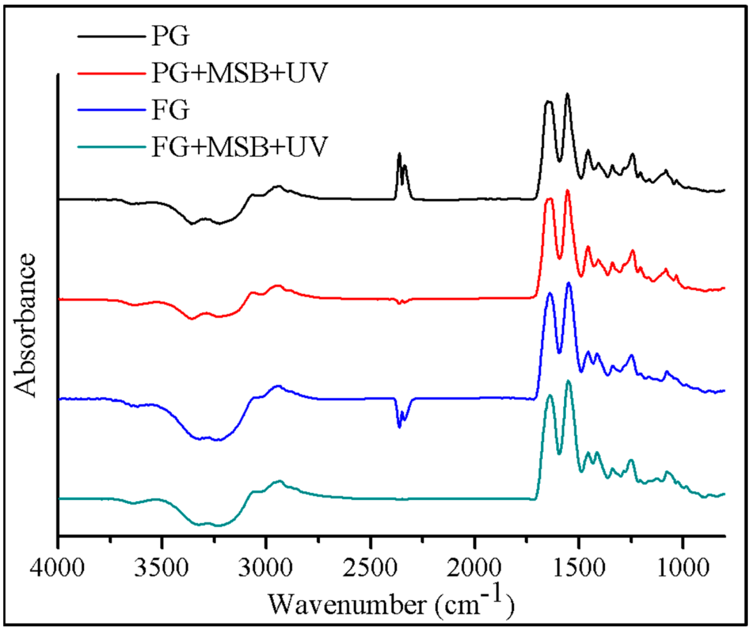

Figure S1. Full ATR-FTIR spectra (4000–800 cm^{-1}) of non-crosslinked and MSB-crosslinked porcine (PG) and fish (FG) gelatin hydrogels at 20 °C. Spectra are vertically offset for clarity.

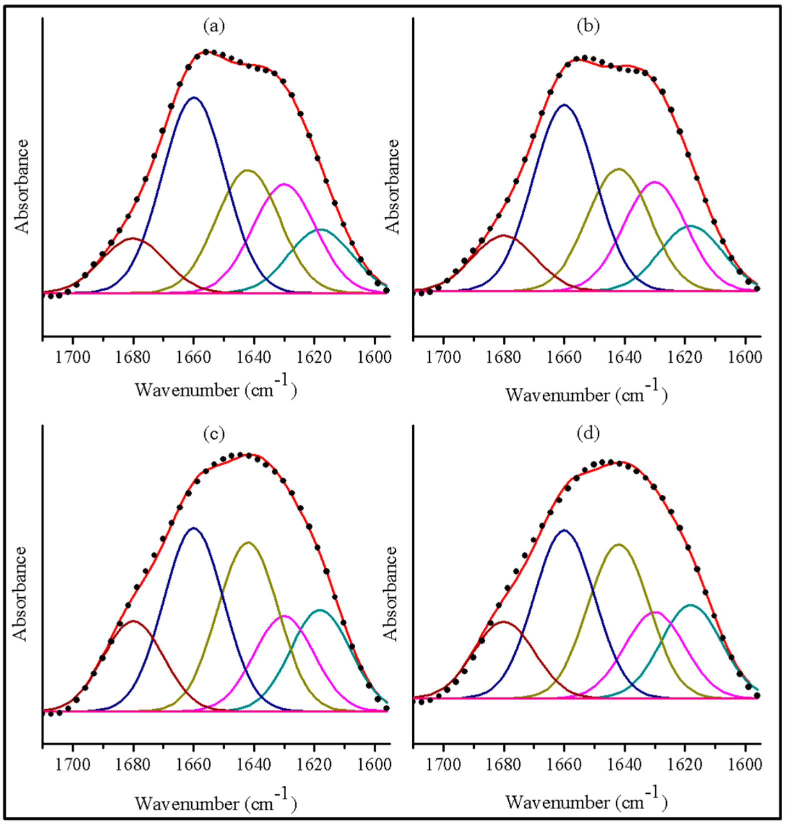

Figure S2. Deconvolution of the Amide-I band into six Gaussian components at 20 °C for (a) PG, (b) PG+MSB+UV, (c) FG, and (d) FG+MSB+UV.

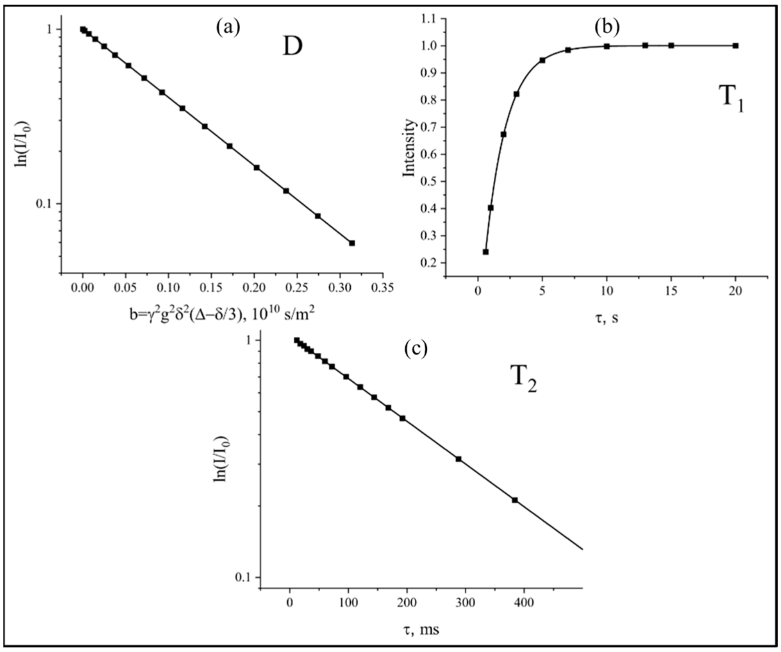

Figure S3. Representative fits used to extract water dynamics parameters from NMR data, shown for fish gelatin (FG) at 5 °C: (a) Diffusion decay, $\ln(I/I_0)$ versus b-factor, fitted with the Stejskal–Tanner equation to obtain the self-diffusion coefficient D; (b) T1 relaxation recovery curve, signal intensity versus relaxation delay τ (s), fitted with an exponential recovery function; (c) T2 relaxation decay measured by the CPMG pulse sequence, $\ln(I/I_0)$ versus echo time τ (ms), fitted with a mono-exponential decay function.

Table S1. Temperature dependence of the free-water spin-lattice relaxation time ($T1free$), the measured population-averaged spin-lattice relaxation time (T_1), and the resulting bound-water fraction (p) for non-crosslinked and MSB-crosslinked porcine (PG) and fish (FG) gelatin hydrogels.

T, °C	$T1$, s				$T1free$	p, %			
	FG	FG+MSB +UV	PG	PG+MSB +UV		FG	FG+MSB +UV	PG	PG+MSB +UV
5	1.66	1.57	1.57	1.28	1.99	2.2	3.0	2.9	6.1
10	1.99	1.79	1.82	1.44	2.35	2.0	3.5	3.3	7.0
15	2.32	2.05	2.11	1.60	2.73	2.0	3.7	3.3	7.9
20	2.72	2.31	2.4	1.78	3.14	1.7	4.0	3.5	8.5
25	2.99	2.57	2.75	2.00	3.58	2.2	4.3	3.3	8.8
30	3.39	2.91	3.16	2.25	4.03	2.1	4.3	3.1	8.8
35	3.78	3.26	3.54	2.5	4.52	2.2	4.3	3.1	8.9
40	4.19	3.54	3.94	2.75	5.03	2.2	4.7	3.1	9.2
45	4.63	3.86	4.30	3.00	5.56	2.2	4.9	3.3	9.5
50	5.20	4.30	4.95	3.27	6.12	2.0	4.7	2.6	9.7
